# Supplementary material for: BitterSweet: Building machine learning models for predicting the bitter and sweet taste of small molecules
Source: Sci Rep. 2019 May 9;9:7155. doi: 10.1038/s41598-019-43664-y (PMC6509165; doi:10.1038/s41598-019-43664-y)
Supplement: Supplementary file 1 — Supplementary Information [file 41598_2019_43664_MOESM1_ESM.pdf]

SUPPLEMENTARY DATA

BitterSweet: Building machine learning models for predicting the bitter and sweet taste of small molecules.

Rudraksh Tuwani, Somin Wadhwa & Ganesh Bagler*

Complex Systems Laboratory, Center for Computational Biology, Indraprastha Institute of Information Technology (IIIT-Delhi), New Delhi, India

*Corresponding Author (ganesh.bagler@gmail.com, bagler@iiitd.ac.in)

Supplementary Figure S1: Percentage of variation captured by the top 10 principal components in Dragon2D, Dragon2D/3D, Canvas, and ChemoPy molecular descriptor sets, for (a) sweet/non-sweet and (b) bitter/non-bitter prediction datasets.

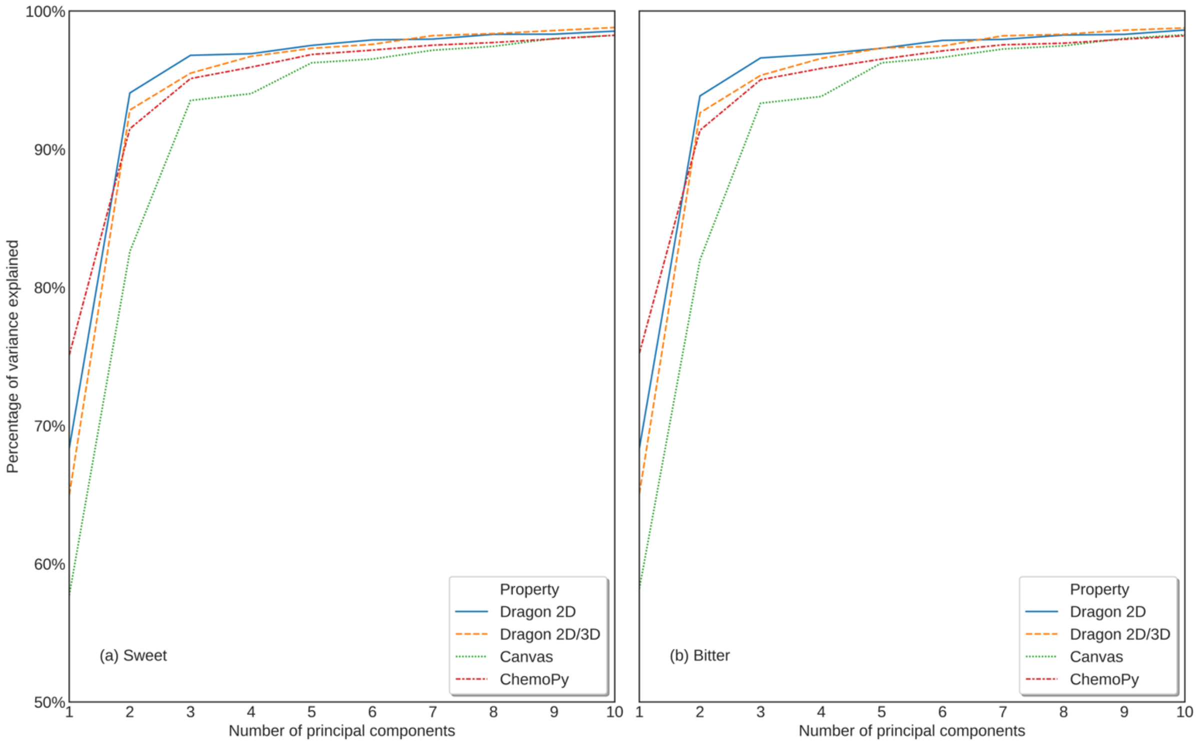

Supplementary Figure S2: Boxplot of importance scores of different ChemoPy descriptors for (a) sweet/non-sweet and (b) bitter/non-bitter prediction.

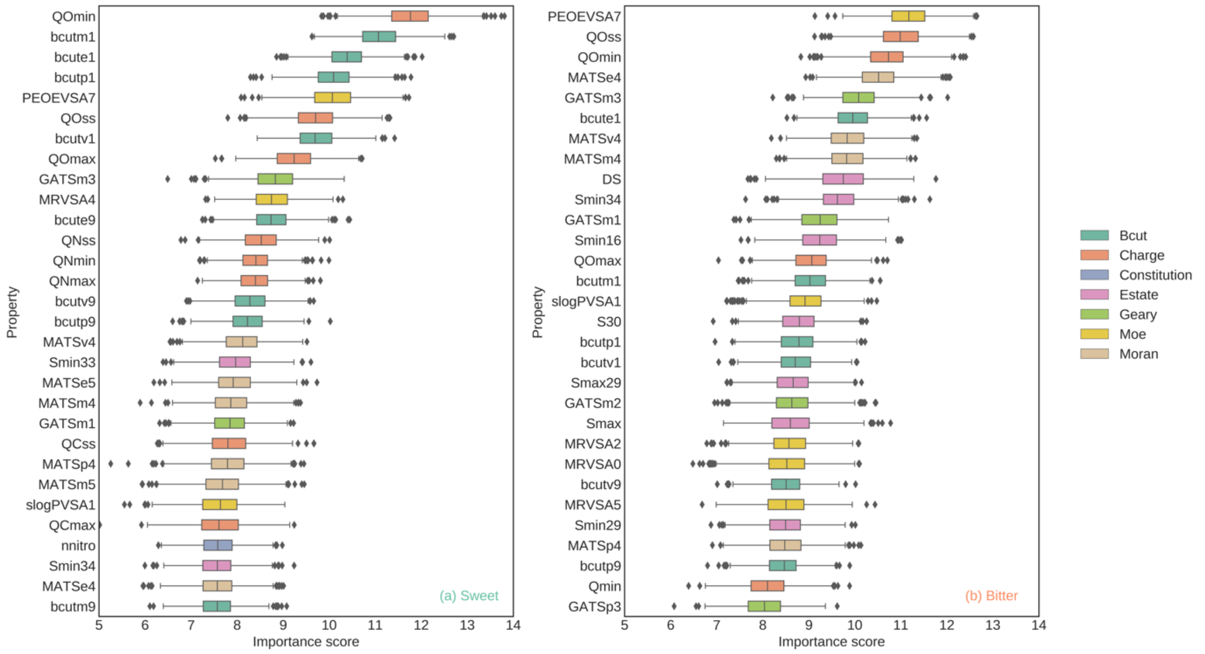

Supplementary Figure S3: Boxplot of importance scores of different Dragon 2D/3D molecular descriptors for (a) sweet/non-sweet and (b) bitter/non-bitter prediction.

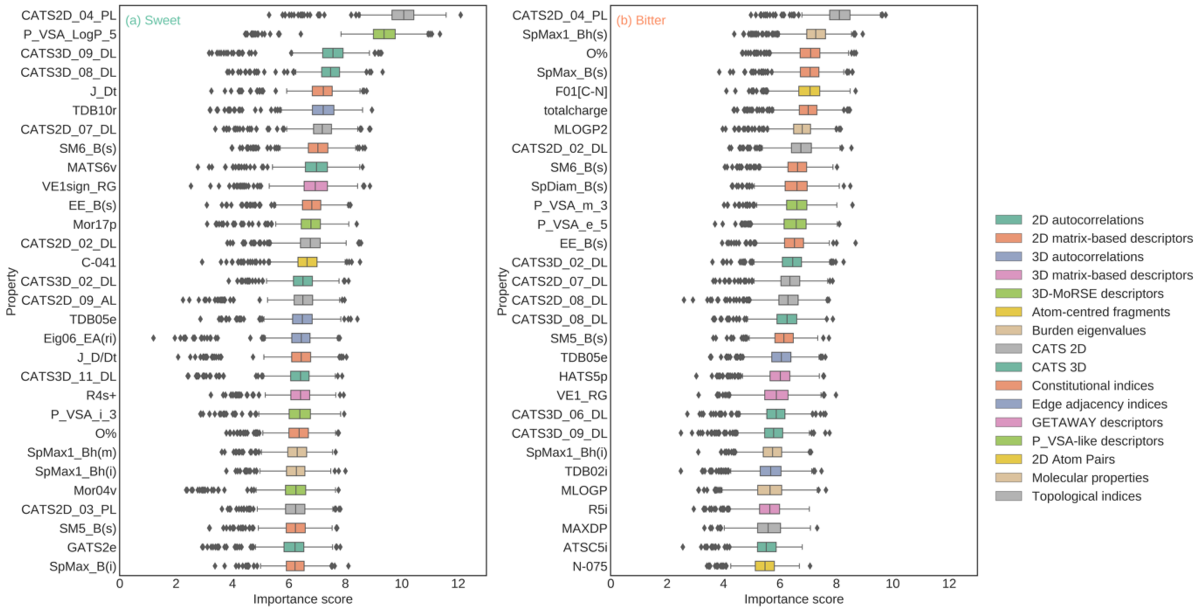

Supplementary Figure S4: Boxplot of importance scores of different Canvas features for (a) sweet/non-sweet and (b) bitter/non-bitter prediction.

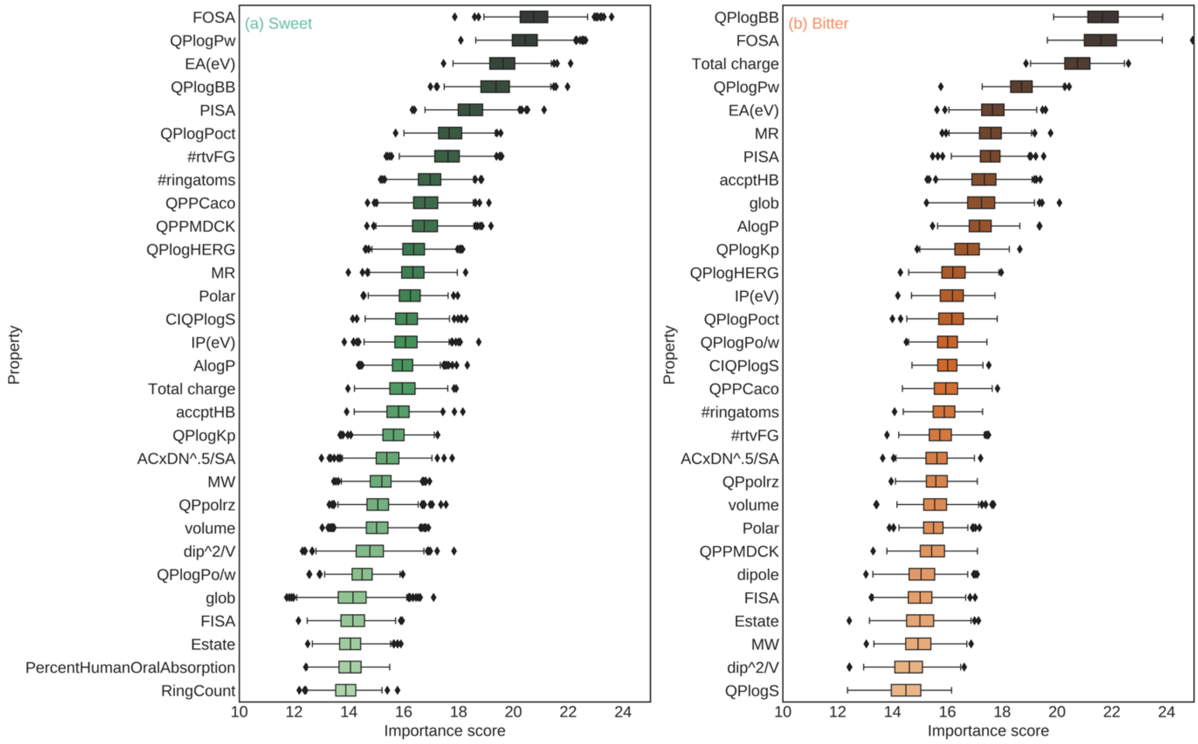

Supplementary Figure S5: Proportion of compounds predicted to be (a) bitter and (b) sweet in specialized chemical sets at different thresholds.

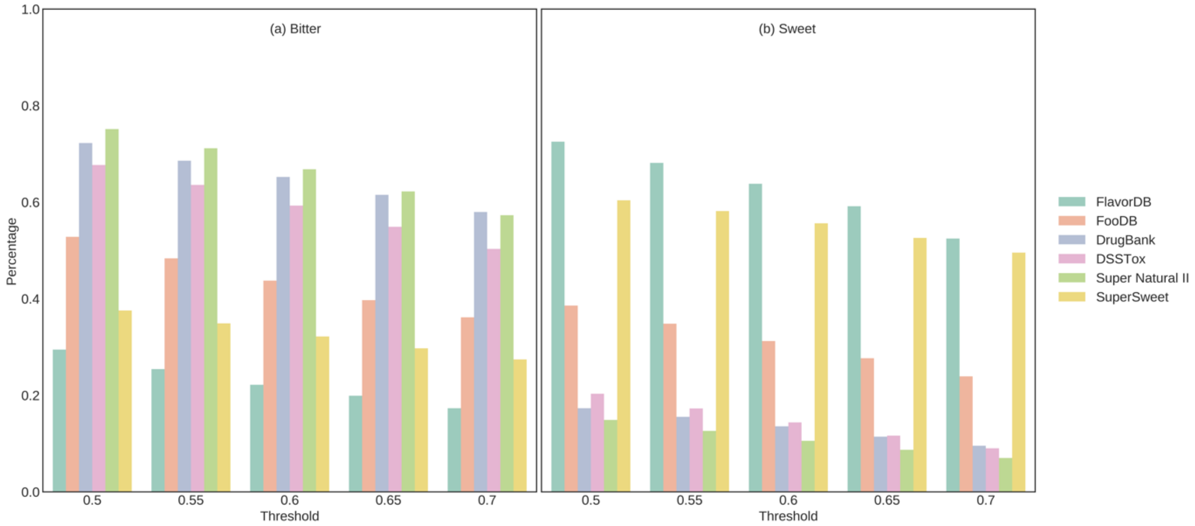

Supplementary Figure S6: Proportion of compounds predicted to be both bitter and sweet in specialized chemical sets at different thresholds.

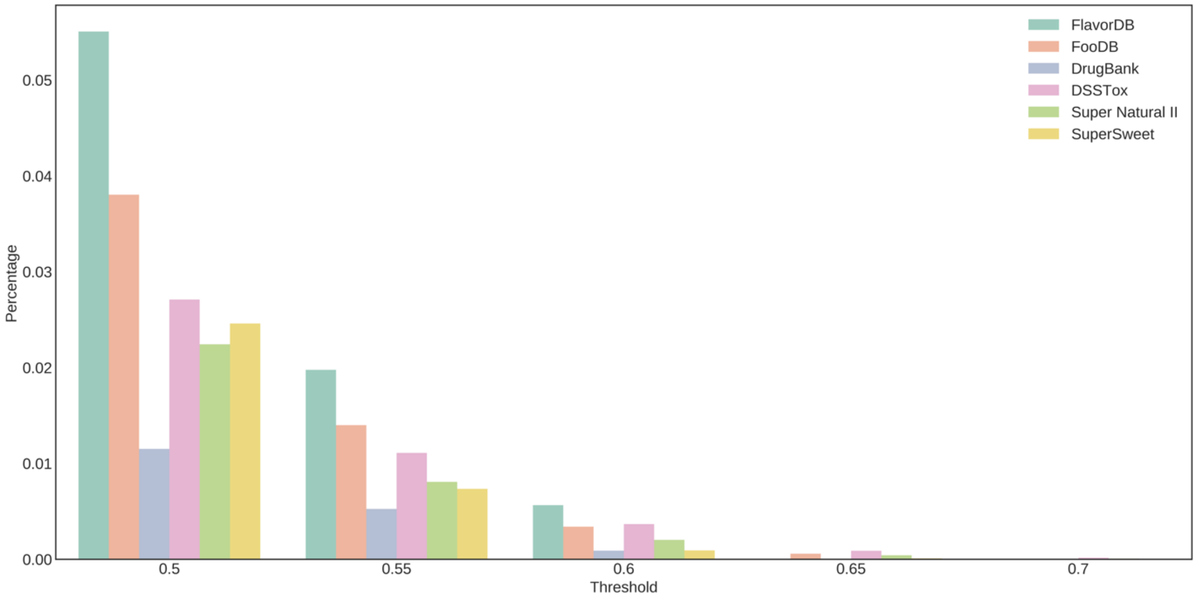

Supplementary Table S1: Number of molecules in training and testing sets for bitter/non-bitter and sweet/non-sweet prediction.

	Type	Taste	Number of molecules
**Bitter**	Training	Bitter	813
		Non-bitter	1444
	Testing	Bitter	95
		Non-bitter	59
**Sweet**	Training	Sweet	1139
		Non-sweet	1066
	Testing	Non-sweet	53
		Sweet	108

Supplementary Table S2: Summary of the different feature sets for building bitter-sweet prediction models.

Software	Description
Canvas	Physicochemical and ADMET (absorption, distribution, metabolism, excretion, and toxicity)
Dragon 7	Extended Connectivity Fingerprints
	2D Molecular Descriptors
	2D/3D Molecular Descriptors
ChemoPy	2D Topological and Structural Features

Supplementary Table S3: Performance of BitterSweet models for sweet/non-sweet prediction.

			Cross-Validation						Test					
Performance Metrics			AUPR	AUROC	F1	NER	Sn	Sp	AUPR	AUROC	F1	NER	Sn	Sp
Molecular Descriptors	Model	Pre-processing												
Canvas	RF	Boruta	0.919	0.910	0.832	0.8380	0.823	0.853	0.899	0.816	0.754	0.7735	0.629	0.918
	RLR	Boruta	0.789	0.801	0.758	0.7510	0.759	0.743	0.882	0.789	0.783	0.7505	0.705	0.796
	AB	Boruta	0.895	0.895	0.826	0.8350	0.794	0.876	0.900	0.837	0.791	0.7710	0.705	0.837
Dragon2D	RF	PCA	0.923	0.919	0.841	0.8480	0.827	0.869	0.924	0.839	0.720	0.7740	0.567	0.981
	RLR	PCA	0.881	0.891	0.829	0.8265	0.836	0.817	0.918	0.832	0.747	0.7655	0.625	0.906
	AB	PCA	0.901	0.895	0.820	0.8305	0.802	0.859	0.928	0.851	0.806	0.7755	0.740	0.811
	RF	Boruta	0.924	0.923	0.847	0.8510	0.835	0.867	0.933	0.863	0.798	0.8130	0.683	0.943
	RLR	Boruta	0.872	0.880	0.824	0.8230	0.837	0.809	0.911	0.812	0.761	0.7750	0.644	0.906
	AB	Boruta	0.923	0.918	0.848	0.8515	0.832	0.871	0.944	0.881	0.829	0.7995	0.769	0.830
Dragon2D/3D	RF	PCA	0.925	0.916	0.849	0.8505	0.840	0.861	0.930	0.843	0.809	0.7905	0.724	0.857
	RLR	PCA	0.889	0.896	0.845	0.8365	0.851	0.822	0.918	0.831	0.804	0.8015	0.705	0.898
	AB	PCA	0.906	0.900	0.819	0.8355	0.769	0.902	0.932	0.845	0.770	0.7985	0.638	0.959
	RF	Boruta	0.929	0.921	0.853	0.8505	0.849	0.852	0.945	0.872	0.798	0.8175	0.676	0.959
	RLR	Boruta	0.842	0.842	0.786	0.7805	0.800	0.761	0.892	0.789	0.757	0.7680	0.638	0.898
	AB	Boruta	0.920	0.918	0.858	0.8560	0.853	0.859	0.950	0.883	0.856	0.8340	0.790	0.878
ChemoPy	RF	PCA	0.918	0.914	0.839	0.8460	0.819	0.873	0.925	0.835	0.731	0.7660	0.592	0.940
	RLR	PCA	0.864	0.868	0.796	0.7950	0.802	0.788	0.887	0.802	0.791	0.7590	0.718	0.800
	AB	PCA	0.894	0.892	0.801	0.8230	0.737	0.909	0.910	0.809	0.762	0.7450	0.670	0.820
	RF	Boruta	0.925	0.922	0.842	0.8495	0.829	0.870	0.933	0.852	0.772	0.8005	0.641	0.960
	RLR	Boruta	0.840	0.852	0.788	0.7875	0.792	0.783	0.877	0.786	0.754	0.7605	0.641	0.880
	AB	Boruta	0.907	0.906	0.819	0.8315	0.797	0.866	0.926	0.838	0.753	0.7805	0.621	0.940
ECFP	RF	None	0.930	0.929	0.862	0.8630	0.869	0.857	0.927	0.837	0.763	0.8020	0.623	0.981
	RLR	None	0.895	0.889	0.815	0.8160	0.813	0.819	0.889	0.760	0.699	0.7545	0.547	0.962
	AB	None	0.923	0.926	0.867	0.8675	0.863	0.872	0.929	0.847	0.806	0.7875	0.726	0.849

Supplementary Table S4: Performance of BitterSweet models for bitter/non-bitter prediction aggregated over different test sets.

			Cross-Validation						Test					
Performance Metrics			AUPR	AUROC	F1	NER	Sn	Sp	AUPR	AUROC	F1	NER	Sn	Sp
Molecular Descriptors	Model	Pre-processing												
Canvas	RF	Boruta	0.774	0.840	0.708	0.7635	0.728	0.799	0.908	0.864	0.824	0.7865	0.800	0.773

	RLR	Boruta	0.617	0.709	0.595	0.6670	0.694	0.640	0.819	0.740	0.737	0.6620	0.733	0.591
	AB	Boruta	0.804	0.849	0.722	0.7830	0.739	0.827	0.907	0.866	0.824	0.7995	0.781	0.818
Dragon2D	RF	PCA	0.805	0.855	0.715	0.7630	0.727	0.799	0.911	0.857	0.813	0.7535	0.825	0.682
	RLR	PCA	0.781	0.847	0.722	0.7735	0.797	0.750	0.915	0.850	0.817	0.8025	0.757	0.848
	AB	PCA	0.805	0.863	0.737	0.7915	0.723	0.860	0.912	0.868	0.849	0.7930	0.874	0.712
	RF	Boruta	0.814	0.864	0.747	0.7925	0.787	0.798	0.907	0.855	0.830	0.7970	0.806	0.788
	RLR	Boruta	0.774	0.825	0.697	0.7505	0.758	0.743	0.909	0.836	0.816	0.7900	0.777	0.803
	AB	Boruta	0.812	0.860	0.745	0.7920	0.778	0.806	0.908	0.882	0.837	0.7990	0.825	0.773
Dragon2D/3D	RF	PCA	0.808	0.857	0.706	0.7670	0.696	0.838	0.919	0.874	0.869	0.8055	0.914	0.697
	RLR	PCA	0.797	0.862	0.739	0.8020	0.831	0.773	0.909	0.845	0.833	0.7695	0.857	0.682
	AB	PCA	0.738	0.787	0.645	0.7255	0.626	0.825	0.909	0.851	0.842	0.7980	0.838	0.758
	RF	Boruta	0.784	0.851	0.726	0.7815	0.753	0.810	0.904	0.853	0.833	0.7855	0.829	0.742
	RLR	Boruta	0.770	0.840	0.680	0.7530	0.773	0.733	0.902	0.844	0.829	0.7780	0.829	0.727
	AB	Boruta	0.782	0.854	0.714	0.7745	0.696	0.853	0.921	0.873	0.817	0.8110	0.743	0.879
ChemoPy	RF	PCA	0.797	0.852	0.698	0.7540	0.719	0.789	0.927	0.880	0.838	0.8190	0.790	0.848
	RLR	PCA	0.743	0.821	0.685	0.7505	0.756	0.745	0.931	0.884	0.856	0.8180	0.848	0.788
	AB	PCA	0.778	0.852	0.713	0.7775	0.705	0.850	0.909	0.869	0.833	0.7855	0.829	0.742
	RF	Boruta	0.803	0.852	0.725	0.7705	0.761	0.780	0.917	0.874	0.844	0.7950	0.848	0.742
	RLR	Boruta	0.744	0.808	0.669	0.7310	0.751	0.711	0.905	0.846	0.818	0.7390	0.857	0.621
	AB	Boruta	0.786	0.850	0.716	0.7665	0.705	0.828	0.897	0.873	0.837	0.8065	0.810	0.803
ECFP	RF	None	0.791	0.837	0.668	0.7240	0.614	0.834	0.906	0.861	0.850	0.7870	0.892	0.682
	RLR	None	0.757	0.815	0.693	0.7430	0.810	0.676	0.846	0.789	0.840	0.7615	0.902	0.621
	AB	None	0.823	0.860	0.721	0.7725	0.762	0.783	0.926	0.892	0.846	0.8055	0.838	0.773

Supplementary Table S5: Performance of BitterSweet models for bitter/non-bitter prediction on the Phyto-Dictionary test set.

Molecular Descriptors	Model	Pre-processing	Sn	Sp	F1	AUPR	AUROC
Canvas	RF	Boruta	0.980	0.76	0.932	0.959	0.931
	RLR	Boruta	0.776	0.76	0.817	0.939	0.913
	AB	Boruta	0.939	0.76	0.911	0.935	0.968
Dragon2D	RF	PCA	0.979	0.76	0.931	0.985	0.972
	RLR	PCA	0.979	0.64	0.904	0.971	0.884
	AB	PCA	0.979	0.84	0.949	0.989	0.979
	RF	Boruta	0.958	0.84	0.939	0.971	0.951
	RLR	Boruta	0.938	0.68	0.891	0.947	0.898
	AB	Boruta	0.979	0.76	0.931	0.978	0.959
Dragon2D/3D	RF	PCA	0.980	0.68	0.914	0.976	0.946
	RLR	PCA	0.959	0.56	0.879	0.950	0.886
	AB	PCA	0.939	0.80	0.920	0.982	0.953
	RF	Boruta	0.959	0.76	0.922	0.962	0.931
	RLR	Boruta	0.980	0.68	0.914	0.952	0.913
	AB	Boruta	0.959	0.88	0.949	0.985	0.968
ChemoPy	RF	PCA	0.959	0.92	0.959	0.988	0.972
	RLR	PCA	0.939	0.56	0.868	0.944	0.884
	AB	PCA	0.959	0.92	0.959	0.990	0.979
	RF	Boruta	0.959	0.84	0.940	0.975	0.951
	RLR	Boruta	0.898	0.68	0.871	0.952	0.898
	AB	Boruta	0.959	0.88	0.949	0.980	0.959
ECFP	RF	None	0.979	0.84	0.949	0.972	0.946
	RLR	None	0.958	0.64	0.893	0.937	0.886
	AB	None	0.959	0.84	0.940	0.970	0.953

Supplementary Table S6: Performance of BitterSweet models for bitter/non-bitter prediction on the UNIMI test set.

Molecular Descriptors	Model	Pre-processing	Sn	Sp	F1	AUPR	AUROC
Canvas	RF	Boruta	0.826	0.727	0.745	0.810	0.845
	RLR	Boruta	0.870	0.364	0.625	0.626	0.664
	AB	Boruta	0.739	0.818	0.739	0.844	0.875
Dragon2D	RF	PCA	0.826	0.576	0.679	0.667	0.794
	RLR	PCA	0.609	0.970	0.737	0.848	0.842
	AB	PCA	0.783	0.667	0.692	0.557	0.741
	RF	Boruta	0.783	0.727	0.720	0.695	0.814
	RLR	Boruta	0.696	0.879	0.744	0.854	0.856
	AB	Boruta	0.739	0.758	0.708	0.772	0.829
Dragon2D/3D	RF	PCA	0.913	0.636	0.750	0.687	0.817
	RLR	PCA	0.826	0.424	0.623	0.659	0.740
	AB	PCA	0.783	0.818	0.766	0.850	0.888
	RF	Boruta	0.826	0.697	0.731	0.690	0.802
	RLR	Boruta	0.913	0.727	0.792	0.844	0.896
	AB	Boruta	0.565	0.879	0.650	0.768	0.830

**ChemoPy**	**RF**	**PCA**	0.478	0.848	0.564	0.694	0.790
	**RLR**	**PCA**	0.957	0.515	0.721	0.797	0.840
	**AB**	**PCA**	0.913	0.545	0.712	0.634	0.727
	**RF**	**Boruta**	0.957	0.636	0.772	0.716	0.829
	**RLR**	**Boruta**	1.000	0.515	0.742	0.774	0.831
	**AB**	**Boruta**	0.739	0.727	0.694	0.741	0.829
**ECFP**	**RF**	**None**	0.957	0.515	0.721	0.590	0.711
	**RLR**	**None**	1.000	0.515	0.742	0.547	0.688
	**AB**	**None**	0.913	0.697	0.778	0.783	0.848

Supplementary Table S7: Performance of BitterSweet models for bitter/non-bitter prediction on the Bitter-New test set.

Molecular Descriptors	Model	Pre-processing	Sn	Sp	F1	AUPR	AUROC
**Canvas**	**RF**	**Boruta**	0.652	-	0.789	1.0	-
	**RLR**	**Boruta**	0.565	-	0.722	1.0	-
	**AB**	**Boruta**	0.783	-	0.878	1.0	-
**Dragon2D**	**RF**	**PCA**	0.652	-	0.789	1.0	-
	**RLR**	**PCA**	0.609	-	0.757	1.0	-
	**AB**	**PCA**	0.739	-	0.850	1.0	-
	**RF**	**Boruta**	0.652	-	0.789	1.0	-
	**RLR**	**Boruta**	0.652	-	0.789	1.0	-
	**AB**	**Boruta**	0.696	-	0.821	1.0	-
**Dragon2D/3D**	**RF**	**PCA**	0.913	-	0.955	1.0	-
	**RLR**	**PCA**	0.739	-	0.850	1.0	-
	**AB**	**PCA**	0.565	-	0.722	1.0	-
	**RF**	**Boruta**	0.739	-	0.850	1.0	-
	**RLR**	**Boruta**	0.652	-	0.789	1.0	-
	**AB**	**Boruta**	0.652	-	0.789	1.0	-
**ChemoPy**	**RF**	**PCA**	0.565	-	0.722	1.0	-
	**RLR**	**PCA**	0.826	-	0.905	1.0	-
	**AB**	**PCA**	0.652	-	0.789	1.0	-
	**RF**	**Boruta**	0.652	-	0.789	1.0	-
	**RLR**	**Boruta**	0.696	-	0.821	1.0	-
	**AB**	**Boruta**	0.696	-	0.821	1.0	-
**ECFP**	**RF**	**None**	0.739	-	0.850	1.0	-
	**RLR**	**None**	0.826	-	0.905	1.0	-
	**AB**	**None**	0.652	-	0.789	1.0	-
